# Effects and Safety of Gyejibongnyeong-Hwan on Dysmenorrhea Caused by Blood Stagnation: A Randomized Controlled Trial

**DOI:** 10.1155/2013/424730

**Published:** 2013-09-26

**Authors:** Jeong-Su Park, Sunju Park, Chun-Hoo Cheon, Seong-Cheon Jo, Han Baek Cho, Eun-Mee Lim, Hyung Ho Lim, Bo-Hyoung Jang, Yong-Cheol Shin, Seong-Gyu Ko

**Affiliations:** ^1^Center for Clinical Research and Drug Development, College of Korean Medicine, Kyung Hee University, Seoul 130-701, Republic of Korea; ^2^Department of Preventive Medicine, College of Korean Medicine, Kyung Hee University, Seoul 130-701, Republic of Korea; ^3^Department of Preventive Medicine, College of Korean Medicine, Daejeon University, Daejeon 300-716, Republic of Korea; ^4^Department of Obstetrics and Gynecology, College of Korean Medicine, Won-Kwang University, Iksan 570-749, Republic of Korea; ^5^Department of Obstetrics and Gynecology, College of Korean Medicine, Gachon University, Seongnam 461-701, Republic of Korea; ^6^Department of Korean Medicine Rehabilitation, College of Korean Medicine, Gachon University, Seongnam 461-701, Republic of Korea

## Abstract

*Objective*. This study was a multicenter, randomized, double-blind, and controlled trial with two parallel arms: the GJBNH group and the placebo group. This trial recruited 100 women aging 18 to 35 years with primary dysmenorrhea caused by blood stagnation. The investigational drugs, GJBNH or placebo, were administered for two menstrual periods (8 weeks) to the participants three times per day. The participants were followed up for two menstrual cycles after the administration. *Results*. The results were analyzed by the intention-to-treat (ITT) dataset and the per-protocol (PP) dataset. In the ITT dataset, the change of the average menstrual pain VAS score in the GJBNH group was statistically significantly lower than that in the control group. Significant difference was not observed in the SF-MPQ score change between the GJBNH group and the placebo group. No significant difference was observed in the PP analyses. In the follow-up phase, the VAS scores of the average menstrual pain and the maximum menstrual pain continually decreased in the placebo group, but they increased in the GJBNH group. *Conclusion*. GJBNH treatment for eight weeks improved the pain of the dysmenorrhea caused by blood stagnation, but it should be successively administered for more than two menstrual cycles. *Trial Registration*. This trial is registered with Current Controlled Trials no. ISRCTN30426947.

## 1. Background

Dysmenorrhea is a common medical complaint in reproductive women worldwide. The prevalence varies from 45% to 95% depending on the definition [1]. In Korea, 78.3% of all adolescent girls have dysmenorrhea during their menstrual periods [2]. 

The first option for the treatment of dysmenorrhea is an over-the-counter drug, such as Ibuprofen, Naproxen, and Mefenamic [3, 4]. However, these medications have not been effective in 20% to 25% of women. Some adverse events such as digestive disorders were reported [5]. 

Alternative therapies were reported to treat primary dysmenorrhea. These treatments include acupuncture [6], auricular acupressure [7], infrared-emitting sericite belt [8], and single oral dose of vitamin D [9] and vitamin E [10]. The effectiveness was observed in the treatments, but most studies were pilot trials with small sample sizes. Large-scale clinical trials are needed to clarify the efficacy. 

In Korean Medicine, the main factor causing menstrual abdominal pain is blood stagnation. If the flow of blood or *qi* is interrupted, it may cause pain. The signs of blood stagnation are being easily bruised, tender abdominal pain, loaf in the menstrual blood, and so forth. Gyejibongnyeong-hwan (GJBNH) is one of the most popular Korean Medicine formulas for periodical pain due to dysmenorrhea. GJBNH fluidifies blood to induce smooth blood flow and reduce pain [6]. However, the effectiveness was reported mostly in the form of case reports or noncontrolled one-group clinical trials [11, 12]. The well-designed clinical trial is mandatory to prove the effect of GJBNH in primary dysmenorrhea.

The purpose of this trial is to identify the efficacy of GJBNH in dysmenorrhea caused by blood stagnation.

## 2. Methods

### 2.1. Hypothesis

The hypothesis was that GJBNH would reduce menstrual pain more effectively than placebo after taking the intervention—the GJBNH or the placebo—for two menstrual cycles.

### 2.2. Design

**Figure 1:**
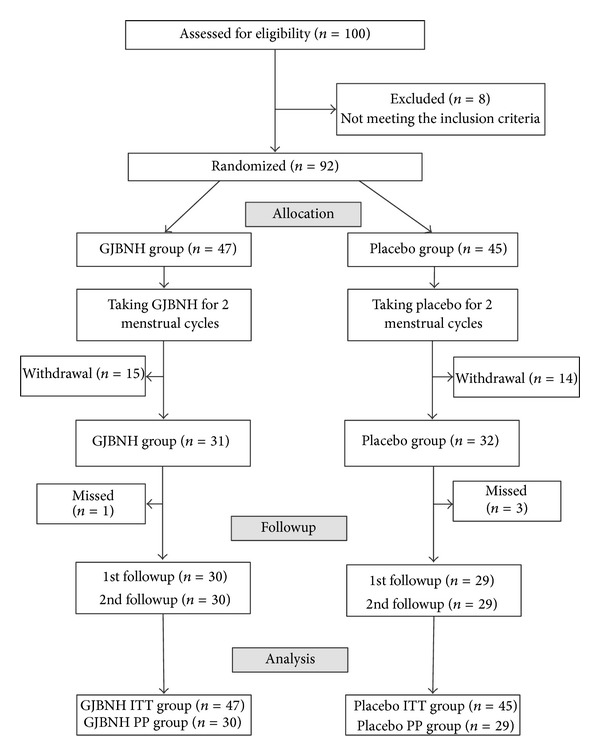
Study flowchart.

This was a multicenter, randomized, double-blind, parallel group, and placebo-controlled phase IV trial. This study was conducted from June 2009 to October 2012. Three investigational sites involved the trial: Korean Medicine Obstetrics and Gynecology Clinic of Kyung Hee Medical Center in Seoul, Obstetrics and Gynecology Clinic of Won-Kwang Korean Medicine Hospital in Gunpo, and Obstetrics and Gynecology Clinic of Gil Korean Medical Hospital of Gachon in Incheon, Republic of Korea. The institutional review boards (IRBs) of three investigational sites had approved the trial before the participants recruitment. Participants were treated as outpatients in these sites. The first visit was the screening. Participants were screened for entry into the trial. Medical, medication, and gynecologic histories were obtained. The second visit was the baseline. At the baseline, the participants were randomly assigned into two groups: the GJBNH group or the placebo group. Eligible participants had taken the investigational drugs for eight weeks. The 3rd visit was after one menstrual cycle from the baseline, and the 4th visit was after two menstrual cycles from the baseline. The 5th visit was after one menstrual period from the 4th visit, and the 6th visit was after two menstrual periods from the 5th visit. At the routine visit, patients returned to the clinic for assessment of the clinical improvement. The trial was conducted over five menstrual cycles. The treatment phase was after two menstrual periods from the baseline (about eight weeks). The participants were followed up three menstrual cycles after the treatment phase.  Figure 1 is a flow diagram of this trial. 

### 2.3. Participants

This trial recruited 100 women aging 18 to 35 years with primary dysmenorrhea. We used the 100 mm visual analogue scale (VAS) to measure the menstrual pain. The women enrolled were those whose pain intensity was more than 60 mm. Two Korean Medicine gynecology specialists diagnosed the participants to determine whether dysmenorrhea was caused by blood stagnation or not. 

 Inclusion criteria were for those with a period cycle of 30 ± 3 days during the last 3 months, for those with a VAS score over 60 mm of VAS at screening, and for those diagnosed with blood stagnation. 

 Exclusion criteria were for those having major neuropsychiatric disorders, planning to have a baby, or taking antidepressant, antiserotonin, barbiturate, or psychotropic drugs. Other inclusion criteria and exclusion criteria were described in the study protocol [13]. 

### 2.4. Intervention

Gyejibongnyeong-hwan (GJBNH) is one of the Korean Medicine formulas for dysmenorrhea caused by blood stagnation. The participants had taken the investigational drug, GJBNH or placebo, three times per day for two menstrual periods (eight weeks). GJBNH consists of *Cinnamomiramulus, Poria, Moutan cortex, Persicae semen, *and *Paeoniae radix*. The placebo medicine was made of lactose, corn starch, and food coloring and had a similar appearance, shape, weight, taste, and color as GJBNH. As rescue medication, ten pain-killer pills were provided during each treatment cycle. 

### 2.5. Randomization

Participants were divided into two groups at Visit 2. The randomization process was commissioned to the independent institution. The random number was produced by a computer random number generator. The central web-site was used to perform the randomization procedure. The investigators, participants, and monitors were blinded to the study purpose and hypothesis. 

### 2.6. Outcomes

The primary outcome was the change in the visual analogue scale (VAS) of the average menstrual pain after the baseline (Visit 2) and after the treatment (Visit 4). The secondary outcome measures included the VAS (the maximum pain during the menstrual period) and the Short-Form McGill Pain Questionnaire (SF-MPQ) [14]. 

### 2.7. Statistical Analyses

We carried out efficacy analyses on ITT (intention-to-treat: all were randomly assigned participants) and PP (per-protocol: participants completed the trial without any protocol violations). For ITT analysis, missing data were imputed by last-observation carried forward (LOCF) method. We used SPSS version 20.0 (IBM, Inc., Chicago, IL, USA) to perform the data analysis. The descriptive statistics were used to compare the demographic characteristics. We performed Student's *t*-test to evaluate the efficacy of GJBNH in the VAS and the SF-MPQ. For categorical outcome variables, the Chi-square test was used to test the difference. The significance level was *P* = 0.05. 

### 2.8. Ethical Consideration

The trial is conducted according to the Declaration of Helsinki 2008 and/or the regulations of the “Good Clinical Practice” principles of the Korea Food & Drug Administration. 

The institutional review boards (IRBs) have approved this clinical trial at all investigational sites before the participants recruitment. The reference numbers are KOMC IRB 2008-07 (IRB of Kyung Hee Oriental Medical Center approved it on the 18th of August 2008), WONSBHB IRB 2009-02 (IRB of Won-Kwang University Sanbon Oriental Medical Center approved it on the 24th of February 2009), and 09-101 (IRB of Kyungwon Gil Oriental Medical Hospital approved it on the 2nd of February 2009). Prior to undertaking any study-related procedures, all participants were fully informed and signed consent forms. 

## 3. Results

A total of 100 women were screened. Eight women did not meet the inclusion criteria, and 92 participants were enrolled. Forty-seven subjects were allocated to the GJBNH group, and forty-five subjects were allocated to the placebo arm. After the eight weeks of treatment, 15 participants in the GJBNH group and 14 patients in the control group were dropped out. Two of the most common reasons for dropout were the irregular menstrual periods, under 27 days and over 33 days, and being due to compliance. Adverse events were rare. The most common compliant was the digestion problem. 

The demographic characteristics of the two groups are described in Table 1. The mean age at randomization for the GJBNH group was 23.36 years, and it was 23.76 years for the control group. The mean Short-Form McGill Pain Questionnaire at baseline for the GJBNH group was 21.23, and it was 22.98 for the placebo group. The mean VAS scores of the average menstrual pain were 7.05 and 6.86 for the GJBNH group and the placebo group, respectively. The mean VAS of the maximum menstrual pain of the GJBNH group was 7.74, and that of the control group was 7.65. There was no statistical significant difference between the two groups in most of the variables assessed at the baseline.

In the ITT analysis, after the eight weeks of treatment, the change of the average menstrual pain VAS score in the GJBNH group was significantly lower than that of the control group (GJBNH group 1.75 ± 2.06, control group 0.88 ± 1.64; *P* = 0.027). But the difference was not statistically significant. The VAS scores of the maximum menstrual pain decreased by 1.03 ± 1.84 and 0.44 ± 2.05 in the GJBNH and the placebo groups, respectively. But the difference was not statistically significant (*P* = 0.155). Significant difference was not observed in the SF-MPQ score change between the GJBNH group and the placebo group: 4.11 ± 8.61 and 2.60 ± 8.86 in the GJBNH group and the placebo group, respectively (*P* = 0.410).  Table 2 described the details of the ITT analysis results.

In the PP analysis, after the eight weeks of treatment, the change of the average menstrual pain VAS score in the GJBNH group was lower than that of the control group (GJBNH group 1.99 ± 2.18, control group 1.09 ± 1.84; *P* = 0.094). But the difference was not statistically significant. The VAS scores of the maximum menstrual pain decreased by 1.26 ± 2.07 and 0.47 ± 2.44 in the GJBNH and the placebo groups, respectively. But the difference was not statistically significant (*P* = 0.188). Significant difference was not observed in the SF-MPQ score change between the GJBNH group and the placebo group: 5.03 ± 9.24 and 3.69 ± 9.81 in the GJBNH group and the placebo group, respectively (*P* = 0.590). The details of the PP analysis were shown in Table 3. 

**Figure 2:**
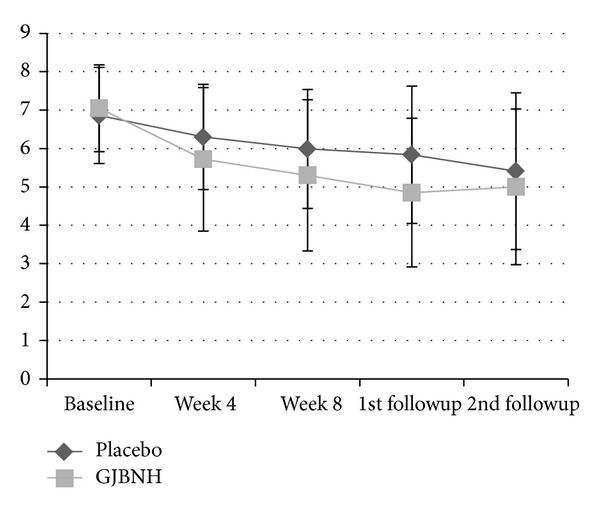
The trend of the GJBNH group and the control group in the VAS of the average menstrual pain—intention-to-treat (ITT) analysis.

**Figure 3:**
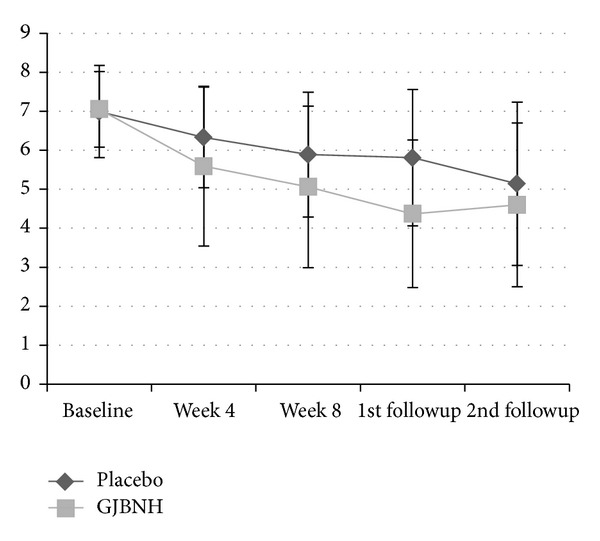
The trend of the GJBNH group and the control group in the VAS of the average menstrual pain—per-protocol (PP) analysis.

The effect of GJBNH maintained one cycle of the menstrual period. Figures 2 and 3 showed the tendency of the VAS of the average menstrual pain. The first followup was after one menstrual cycle after the treatment, and the second followup was after three menstrual cycles after the treatment. The score of the GJBNH group was the lowest at the first followup, but it increased at the second followup. The VAS score of the placebo group decreased continually through the trial. The difference at the first followup between the two groups was more considerable than that after the treatment. But, at the second followup, after the two menstrual cycles after the first followup, the difference tendency disappeared.

### 3.1. Safety

A total of 16 adverse events occurred. The adverse events were mild digestive disorder, breast stabbing pain, menstrual cycle shortage, urticaria, diarrhea, skin itchiness, and nausea. There were eight adverse events in the GJBNH group and eight adverse events in the placebo group. The adverse events rates were not statistically significant (*P* = 0.924).  Table 4 showed the details. 

## 4. Discussion

There are several study reports to treat dysmenorrhea. Acupuncture is a recommendable therapy. Acupuncture was as effective as nonsteroidal anti-inflammatory drug (NSAID) therapy [6]. A randomized controlled trial has shown the effectiveness of acupuncture in primary dysmenorrhea [15]. But there were no significant differences between the groups. Other therapies were effective in primary dysmenorrhea, auricular acupressure [7], infrared-emitting sericite belt [8], single oral doses of vitamin D [9] and vitamin E [10], and so forth. But the statistically significant differences were rare. 

This clinical trial aimed to identify the efficacy of GJBNH on dysmenorrhea caused by blood stagnation. The primary outcome was statistically significant in the ITT analysis. But the result was not significant in the PP analysis. But the result did not show the significant difference compared with the placebo. We discussed the reason for not detecting the difference in the PP analysis, and we concluded that it was due to the lack of sample size and the treatment period. 

The first planned sample size was 100 participants. But the enrolled participants were 92 women. It was due to the pandemic break of influenza. In 2008, the influenza A virus subtype had spread in Korea. The enrolment was delayed. Although this study was supported by the National R&D Project, the study due date was inflexible. As a result, statistical power was lower than the planned.

There was the tendency of the pain decrease in the average menstrual pain between the GJBNH group and the placebo group. A decided difference was observed at Visit 4 and the first followup. This implied that GJBNH should be administered over two menstrual cycles and that the effect of GJBNH remains about one menstrual cycle after the cessation. 

A total number of 18 adverse events occurred in this clinical trial. But of all the adverse events were mild and minor. The adverse events occurrence rates were not different between the groups. We considered that GJBNH was safe for clinical use.

This study assessed the other measurement. We analyzed only the primary endpoint and two secondary endpoints. More advanced analysis is needed for the other assessment. 

The missing value imputation was planned by the multiple imputation (MI) method, but we used the LOCF method. The VAS scores continuously decreased throughout the trial, and the LOCF method was a conservative method. The dropout rates between the groups were not significantly different. Therefore, we inferred that the LOCF method was appropriate to analyze the data. 

This study was valuable to show the effect of GJBNH by the randomized controlled trial. But still there were limitations. The most deficient point was the dropout rate. The participants were mostly young women in the university, and the dropout rate was high in the vacation season. The result was significant in the ITT analysis, but the significance was not shown in the PP analysis. We concluded that the lack of sample size was the main reason. 

We expected that the results of this study would contribute to the practical use in the Korean Medicine clinics and the design of the clinical trial of the Korean Medicine for primary dysmenorrhea.

## Figures and Tables

**Table 1:** Baseline demographics of the GJBNH group and the control group.

	GJBNH (*n* = 47)	Control (*n* = 45)	*P* value
	Mean (SD)	Mean (SD)	
Age (years)	23.36 (3.90)	23.76 (3.77)	0.623
Height (cm)	163.21 (5.58)	161.22 (6.21)	0.109
Weight (kg)	55.06 (7.44)	54.36 (7.13)	0.643
BMI (kg/m^2^)	20.62 (2.18)	21.01 (3.33)	0.512
SBP (mmHg)	117.30 (17.08)	116.00 (14.66)	0.699
DBP (mmHg)	69.13 (11.03)	70.36 (13.58)	0.478
Menarche	12.61 (1.18)	12.71 (1.42)	0.710
Interval of cycles (days)	29.62 (1.33)	29.36 (1.51)	0.380
Dysmenorrhea begins (*n*, %)*			0.873
Under 2 years from menarche	17 (36.2)	17 (37.8)	
Over 2 years from menarche	30 (63.8)	28 (62.2)	
Severity of pain (VAS)	7.29 (1.01)	7.42 (1.1)	0.553

*Means the numbers in that domain indicates *n* (%), not mean (SD).

**Table 2:** Comparison of the outcomes of the GJBNH group and the control group (ITT).

Variables	GJBNH group (*n* = 47)	Control group (*n* = 45)	*P* value
	Mean (SD)	Mean (SD)	
Short-Form McGill Pain Questionnaire			
Baseline	21.23 (7.88)	22.98 (7.97)	
After treatment	17.13 (10.37)	20.38 (9.32)	
Difference	4.11 (8.61)	2.60 (8.86)	0.410
Visual analog scale (average pain)			
Baseline	7.05 (1.13)	6.86 (1.25)	
After treatment	5.30 (1.97)	5.98 (1.55)	
Difference	1.75 (2.06)	0.88 (1.64)	0.027
Visual analog scale (maximum pain)			
Baseline	7.74 (1.14)	7.65 (1.40)	
After treatment	6.72 (1.83)	7.20 (1.89)	
Difference	1.03 (1.84)	0.44 (2.05)	0.155

**Table 3:** Comparison of the outcomes of the GJBNH group and the control group (PP).

Variables	GJBNH group (*n* = 30)	Control group (*n* = 29)	*P* value
	Mean (SD)	Mean (SD)	
Short-Form McGill Pain Questionnaire			
Baseline	21.63 (7.67)	24.62 (7.38)	
After treatment	16.60 (9.72)	20.93 (9.83)	
Difference	5.03 (9.24)	3.69 (9.81)	0.590
Visual analog scale (average pain)			
Baseline	7.05 (0.97)	6.99 (1.18)	
After treatment	5.06 (2.08)	5.89 (1.60)	
Difference	1.99 (2.18)	1.09 (1.84)	0.094
Visual analog scale (maximum pain)			
Baseline	7.62 (1.14)	7.57 (1.43)	
After treatment	6.36 (1.90)	7.09 (2.08)	
Difference	1.26 (2.07)	0.47 (2.44)	0.188

**Table 4:** The adverse events of the GJBNH group and the control group.

Event	GJBNH	Placebo
Mild digestive disorder	3	5
Breast stabbing pain	1	0
Menstrual cycle shortage	0	1
Urticaria	1	2
Diarrhea	1	0
Skin itchiness	1	0
Nausea	1	0

	8 (17%)	8 (18%)
